# Comprehensive Physiotherapy Rehabilitation in a Patient With Cerebellar Ataxia and Dysphagia: A Case Report Investigating Symptomatology, Management, and Outcomes

**DOI:** 10.7759/cureus.63839

**Published:** 2024-07-04

**Authors:** Anam R Sasun, Moh'd Irshad Qureshi, Nitika Chavan, Raghumahanti Raghuveer

**Affiliations:** 1 Department of Neuro-Physiotherapy, Ravi Nair Physiotherapy College, Datta Meghe Institute of Higher Education and Research (DMIHER), Wardha, IND

**Keywords:** cerebellar ataxia, dysphagia, proprioceptive neuromuscular facilitation (pnf), neurophysiotherapy rehabilitation, kinesiotaping

## Abstract

A cerebellar infarct occurs when blood flow to the cerebellum, located in the posterior cranial fossa, is disrupted. This diminished blood supply leads to decreased oxygen delivery, resulting in motor and balance control impairments. One prevalent sign of neurodegenerative diseases is dysphagia, which is typically linked to a higher death rate. No systematic and uniform assessment of dysphagia is used in the clinical care environment of individuals with ataxia. Its effect on the quality of life associated with health in patients is little understood. Therefore, this case report works to address dysphagia in cerebellar ataxia. This case report examines the physiotherapy management of a 41-year-old male who had cerebellar ataxia secondary to an infarct in the bilateral cerebellar hemisphere and vermis. The rehabilitation period lasted for six weeks. On examination, the patient had difficulty swallowing and showed symptoms of cerebellar dysfunctions, such as nystagmus, dyssynergia, dysmetria, and dysdiadochokinesia. Neuro-physiotherapy interventions, like conventional physiotherapy, trunk, and pelvis proprioceptive neuromuscular facilitation (PNF), Kinesio taping for dysphagia, interventions to treat gait, balance training interventions, and Frenkel’s exercises were commenced. The outcome measures were evaluated using standardized outcome measures like the Swallowing Quality of Life Scale (SWAL-QOL), Severity of Ataxia Scale (SARA), Trunk Impairment Scale (TIS), Berg Balance Scale (BBS), Barthel Index, and World Health Organization Quality of Life (WHO-QOL). We conclude that a properly structured physiotherapy program subsequently improved the symptoms of patients. Furthermore, it enhanced functional independence, which subsequently improved the patient's quality of life.

## Introduction

Cerebellar ataxia is a group of disorders that manifest as difficulties in precisely controlling movements, resulting in unsteadiness and impaired balance [1]. Cerebellar injuries may alter the neurological networks that control movement coordination, leading to anomalies in motor execution and balance problems [2]. Movement problems and cognitive abnormalities are common symptoms of cerebellar ataxia, which calls for a comprehensive study encompassing imaging and laboratory testing to determine the variety of underlying reasons. Gait instability, limb incoordination, dysarthria, vertigo, and dysphagia are among the signs of cerebellar ataxia [3]. Increased fall risk is directly linked to cerebellar disease's altered gait, which includes wider steps, trunk oscillations, disorganized patterns, and decreased stability [4]. Research on dysphagia in ataxia disorders is limited, particularly regarding its prevalence, consequences, and prognostic significance.

Dysphagia is a common symptom and is generally associated with increased mortality in neurodegenerative disorders. Although dysphagia is a significant complaint in spinocerebellar ataxia (SCA) patients, little research has been done on the frequency of dysphagia and how it relates to other clinical traits [5]. A cerebellar infarct, also known as a cerebellar stroke, occurs in the posterior cranial fossa, affecting the cerebellum. This reduced blood flow leads to a diminished oxygen supply, resulting in motor and balance control deficits [6]. Brain swelling is caused by cerebellar infarctions because of cytotoxic and vasogenic edema. On the other hand, males and middle-aged to older individuals are more likely to experience cerebellar hemorrhages, which account for 9-10% of all intracranial hemorrhages [7].

Although both ataxia and dysphagia may come from multiple causes, effective rehabilitation is critical in managing dysphagia and ataxia. Dysphagia is a cardinal complaint often ignored in rehabilitation. According to a study conducted, about 17% demonstrate dysphagia based on the Swallowing Quality of Life Scale (SWAL-QOL) score [8]. Several functional imaging studies indicate that the cerebellum exhibits activity during voluntary swallowing [9]. The advancement of chronic neurological conditions often correlates with a decline in quality of life. Therefore, this case report aims to explore physiotherapy rehabilitation for a patient with cerebellar ataxia secondary to a cerebellar infarct. It includes interventions for improving static and dynamic trunk balance, dysphagia, coordination, improving the strength of the pelvis and trunk musculature, and postural control, which will further improve quality of life. Kinesio taping was used to treat dysphagia in this patient. Kenzo Kase created the elastic, waterproof, and breathable kinesiology tape (KT) in the 1970s. After use, it can be recoiled and extended to 120-140% of its initial length. In the domains of sports and medicine, it is a commonly employed treatment because KT is elastic and comfortable and permits more range of motion.

## Case presentation

Patient information

A 41-year-old male, previously employed as an electrician, presented at the neuro-physiotherapy outpatient department with complaints of imbalance while walking over the past three months. The imbalance was noted to have a gradual onset and progressive nature. In addition, he reported progressive swaying to the right side over the past two months, noticeable both while sitting and walking. He also reported difficulty swallowing for the past two months. Furthermore, he complained of severe, episodic, sharp shooting headaches, predominantly on the right side, with aggravation upon moving his head from side to side for the past 15 days. Upon reviewing medical history, the computed tomography image report revealed an infarct in the bilateral cerebellar hemisphere and vermis. His personal history revealed a 15-year history of alcohol use.

Clinical examination

After gaining informed consent from the patient, the examination was done. On observation, the patient was mesomorphic. Neurological assessments were done on day 1, week 3, and week 6 of rehabilitation. The central nervous system examination revealed the presence of positive cerebellar signs. The neurological examination is given in Table 1.

**Table 1:** Neurological examination Grade III: able to accomplish the activity, slightly less than normal, speed and steadiness. Grade II: able to accomplish an activity, movements are slow, awkward, and unsteady. Fair: able to maintain balance with support, may require occasional assistance. Poor: Handheld support required, moderate to maximal assistance required.

Examination	Testing	Findings
Higher cortical functions	Consciousness and orientation	The patient is conscious, cooperative, and well-oriented.
Cranial-nerve examination	Oculomotor nerve	Right lateral nystagmus is present in the right eye.
	Vestibulocochlear nerve	Vestibular part affected
	Glossopharyngeal nerve	Absent (difficulty in swallowing)
Sensation examination	Sensory testing	All sensations are intact
Reflex examination	Reflex testing	All superficial and deep reflexes were present
Motor examination	Muscle tone	No increase in muscle tone
	Manual muscle test	Upper limb (bilateral): 4/5 (Oxford grading). Lower limb (bilateral): 3/5 (Oxford grading)
	Tightness	Tightness of tendon Achilles, hamstring, and quadriceps present
Cerebellar signs	Dyssynergia	Positive
	Dysdiadochokinesia	Positive
	Dysmetria (Finger-nose-test)	Positive
	Dysmetria (Heel-shin- test)	Positive
	Nystagmus	Right lateral nystagmus is present in the right eye
	Titubation	Positive
	Speech disturbance	Absent
Coordination examination	Non-equilibrium	Grade III
	Equilibrium	Grade II
Balance	Static balance	Fair
	Dynamic balance	Poor
Gait analysis	Gait parameters	Wide base of support, reduced speed, and shortened stride length while walking

Physiotherapy rehabilitation

**Figure 1:**
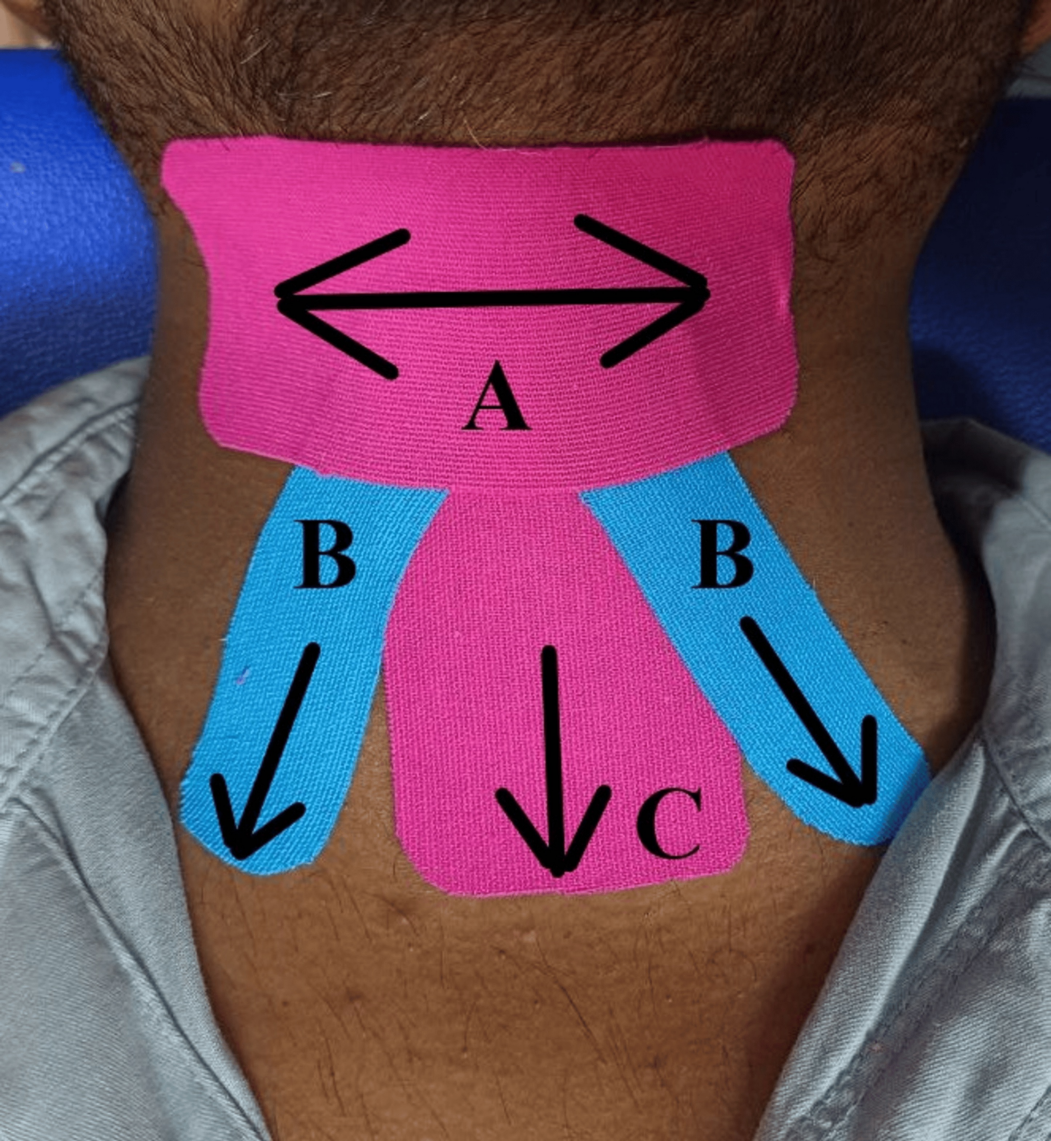
Clinical picture demonstrating application of kinensio taping to the patient C: First, an I-shaped tape was pushed down to the level of the thyroid notch with binding to the sternum. B: The medial superior surface of the clavicle was connected to the hyoid bone via the reverse V-shaped tape. A: To limit movement during swallowing, the hyolaryngeal complex was covered in a horizontal direction.

The physiotherapy rehabilitation was structured according to problem-listing and impairments of the patient. Rehabilitation was commenced for six weeks. The treatment protocol is illustrated in Table 2. Kinesio taping was used to treat dysphagia (Figure 1).

**Table 2:** Physiotherapy rehabilitation of the patient reps: repetitions

Sr No.	Physiotherapy goals	Physiotherapy interventions
1.	Education to the patient	Empower the patient with insights into their existing health condition and introduce the prescribed physiotherapy regimen. Furthermore, facilitate the development of coping strategies to enhance their ability to navigate their current quality of life. These coping mechanisms encompass adept stress management techniques and personalized self-care practices.
2.	To improve swallowing	Horizontal kinesio taping was employed [10]. Effortful swallowing (50 reps), shaker exercises (50 reps, 2 sets each).
3.	To improve static trunk balance	Multi-directional reach-out activities in sitting were commenced.
4.	To improve nystagmus	Cawthrone-Cooksey exercises, gaze stability exercises.
5.	To improve coordination	Sight, sound, and touch were used to compensate for loss. Frenkel's exercises in lying and sitting were commenced (15 repetitions × 3 sets) with rest intervals.
6.	To improve dynamic balance	Trunk and pelvis proprioceptive neuromuscular facilitation techniques.
7.	To Improve Postural control	Pelvic and/post tilt- sitting, lying, standing; sit-ups, sit-ups with rot; four-point kneeling exercises; sitting with upper limb movement were done; bridging with pelvic tilt was commenced. Each activity was done with (10 repetitions × 3 sets) with rest intervals.
9.	To improve gait	Treadmill training (10 mins)

Diagnostic assesment

**Figure 2:**
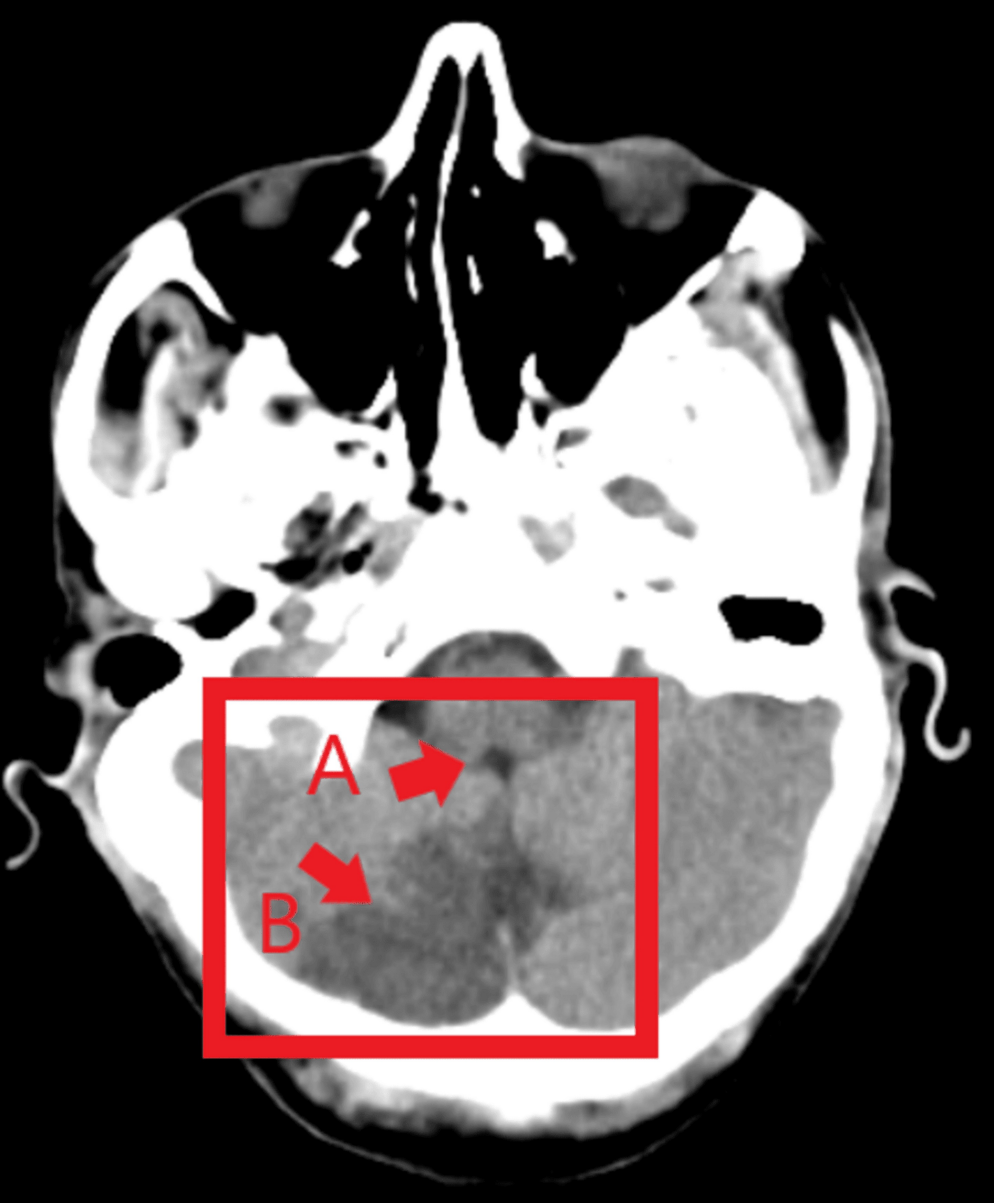
Computed tomography investigation of the patient A: Causing effacement of the fourth ventricle and cisterna magna. B: The presence of an ill-defined, hypodense area in the right cerebellar hemisphere.

There is the presence of an ill-defined hypodense area in the bilateral cerebellar hemisphere (right ≥ left) and vermis, causing effacement of the fourth ventricle, and cisterna magna, causing cerebellar tonsilar herniation (Figure 2).

Outcome measures

The outcome measures showed improvement at the end of rehabilitation (Table 3).

**Table 3:** Outcome measures

Outcome measures	Pre-intervention	Post-intervention
Swallowing Quality of Life (SWAQOL) [11]	46/100	66/100
Berg Balance Scale (BBS) [12]	20/56	46/56
Trunk Impairment Scale (TIS) [13]	16/23	20/23
Barthel Index (BI) [14]	40/100	82/100

## Discussion

This case report describes the positive impact of a six-week neurophysiotherapy rehabilitation. This case report highlights the similarities, differences, and any novel contributions it makes. The similarities are the application of Kinesio taping and functional improvement in the patient. The differences are that this case report specifically compared scores of pre- and post-physiotherapy treatment, and the rehabilitation protocol was structured according to the patient's needs. Significant improvement was observed in six weeks. Numerous outcome measures were used to assess the improvement. The novelty of this case report is that it has included recent evidence for treating physiotherapy. Second, it uses a combination of interventions to treat the condition.

The study aimed to explore physiotherapy rehabilitation for a patient with cerebellar ataxia secondary to cerebellar infarct. It includes interventions for improving static and dynamic trunk balance, dysphagia, coordination, improving the strength of pelvis and trunk musculature, and postural control, which will further improve the quality of life. Current clinical research on the management of these disorders through physiotherapy is limited. Nevertheless, the existing data suggest that an effective treatment approach should prioritize activity-based interventions, with a specific focus on addressing impairments. The diverse prevalence rates of dysphagia in cerebellar ataxia are influenced by both the underlying disease pathology and the methods used for assessment. The understanding of physiotherapy's role in managing cerebellar ataxia remains limited, with a scarcity of literature outlining evidence-based rehabilitation approaches for this condition.

Kinesio-taping mechanisms in dysphagia include several key functions. First, the application of Kinesio taping provides sensory stimulation. It gives the skin and underlying tissues constant sensory stimulation. By increasing proprioception and awareness of the swallowing muscles, this sensory feedback may improve coordination and patterns of muscular activation. Second, it activates muscle by applying tape in a particular direction, which subsequently decreases tension in the muscle. Third, it improves lymphatic drainage. This case report paves the way for new physiotherapy interventions for managing complications of cerebellar ataxia. Dysphagia (oropharyngeal dysphagia) manifests as coughing, choking, food sticking, drooling, prolonged mealtimes, and weight loss, with significant long-term psychosocial effects on the quality of life [15].

According to Sasun et al., the trunk facilitates smoother movement throughout the body. Patients with cerebellar ataxia show greater trunk movements, which reflect a lack of coordination between the segments of the body. The goal of trunk stabilization exercise is to improve muscular control, which is necessary for bracing the trunk against both internal and external stresses. The transversus abdominis muscle has been the focus of rehabilitation programs for assessment and training, despite the fact that all abdominal muscles contribute to spinal stability. The enhancement of joint proprioception through trunk and pelvis proprioceptive neuromuscular facilitation leads to an improvement in pelvic control, which is crucial for maintaining trunk control and balance. Trunk stabilization is essential for aiding upper and lower extremity actions, managing loads effectively, and safeguarding the spinal cord. In dynamic movements, the muscles of the trunk serve as a supportive corset, offering both stability and mobility [16]. Patient-centered outcomes and quality-of-life measures are vital for managing cerebellar ataxia because they focus on the individual experiences and comprehensive needs of patients. These measurements take into account social, emotional, and physical factors to enable individualized and comprehensive care. Advancements in these domains augment physical capabilities, psychological welfare, and interpersonal relationships. Patients have improved long-term health and treatment plan adherence when these varied demands are met. In general, concentrating on the quality of life greatly improves the general well-being of people with cerebellar ataxia.

Jung et al. suggested a study regarding the effects of Kinesio taping in dysphagia. In this study, the tape's elasticity was leveraged to apply loading to the swallowing muscles, prompting patients to exert greater effort during swallowing. This increased effort leads to heightened muscle activation in the tongue and suprahyoid muscles, as overcoming the tape's resistance requires vigorous swallowing, known to effectively strengthen these muscles [17]. Ayvat et al. (2021) conducted a study highlighting the effects of exergame training on individuals with ataxia. Their findings underscored the supplementary advantages of exergame training in enhancing postural control [18]. According to Keagea et al., identifying dysphagia earlier in the rehabilitative phase can be mitigated and optimal health and quality of life can be promoted [19]. Significant improvements in static and dynamic trunk balance, dysphagia, coordination, strength of pelvis and trunk musculature, and postural control were seen.

The strength of this case report is that it provides important clinical insights into the patient's rehabilitation and outcomes. It paves the way for clinical therapists to implement a day-to-day treatment protocol. The limitation of the study is that it underscores the importance of conducting larger-scale studies and research focusing on ataxia and trunk and pelvis proprioceptive neuromuscular facilitation, and Kinensio taping in improving dysphagia. Moreover, the development of care plans for the ataxic population in the future should happen more often.

## Conclusions

The case study concludes by suggesting a notable enhancement in the overall functional status of patients observed over the subsequent six weeks. The clinical implications are as follows: This case report highlights the importance of Kinesio taping in treating dysphagia in cerebellar ataxia and underscores the importance of implementing trunk proprioceptive neuromuscular facilitation exercises. Moreover, this case report has included recent physiotherapy evidence for treating cerebellar ataxia. Looking ahead, our research indicates the potential inclusion of this exercise protocol in the rehabilitation regimen for individuals with ataxia.

## References

[1] Barbuto S, Kuo SH, Winterbottom L, Lee S, Stern Y, O'Dell M, Stein J (2023). Home aerobic training for cerebellar degenerative diseases: a randomized controlled trial. Cerebellum.

[2] Ilg W, Timmann D (2013). Gait ataxia--specific cerebellar influences and their rehabilitation. Mov Disord.

[3] Rossi M, Perez-Lloret S, Doldan L (2014). Autosomal dominant cerebellar ataxias: a systematic review of clinical features. Eur J Neurol.

[4] Earhart GM, Bastian AJ (2001). Selection and coordination of human locomotor forms following cerebellar damage. J Neurophysiol.

[5] Yang CY, Lai RY, Amokrane N (2020). Dysphagia in spinocerebellar ataxias type 1, 2, 3 and 6. J Neurol Sci.

[6] Kase CS, Norrving B, Levine SR, Babikian VL, Chodosh EH, Wolf PA, Welch KM (1993). Cerebellar infarction. Clinical and anatomic observations in 66 cases. Stroke.

[7] Amar AP (2012). Controversies in the neurosurgical management of cerebellar hemorrhage and infarction. Neurosurg Focus.

[8] Rönnefarth M, Hanisch N, Brandt AU, Mähler A, Endres M, Paul F, Doss S (2020). Dysphagia affecting quality of life in cerebellar ataxia—a large survey. Cerebellum.

[9] Suzuki M, Asada Y, Ito J, Hayashi K, Inoue H, Kitano H (2003). Activation of cerebellum and basal ganglia on volitional swallowing detected by functional magnetic resonance imaging. Dysphagia.

[10] Park JS, Jung YJ, Kim HH, Lee G (2020). A novel method using kinesiology taping for the activation of suprahyoid muscles in healthy adults: a preliminary research. Dysphagia.

[11] McHorney CA, Bricker DE, Robbins J, Kramer AE, Rosenbek JC, Chignell KA (2000). The SWAL-QOL outcomes tool for oropharyngeal dysphagia in adults: II. Item reduction and preliminary scaling. Dysphagia.

[12] Winser SJ, Smith C, Hale LA, Claydon LS, Whitney SL (2015). Balance outcome measures in cerebellar ataxia: a Delphi survey. Disabil Rehabil.

[13] Verheyden G, Nieuwboer A, Mertin J, Preger R, Kiekens C, De Weerdt W (2004). The Trunk Impairment Scale: a new tool to measure motor impairment of the trunk after stroke. Clin Rehabil.

[14] Duffy L, Gajree S, Langhorne P, Stott DJ, Quinn TJ (2013). Reliability (inter-rater agreement) of the Barthel Index for assessment of stroke survivors: systematic review and meta-analysis. Stroke.

[15] Rivelsrud MC, Kirmess M, Hartelius L (2024). Cultural adaptation and validation of the Norwegian version of the swallowing quality of life questionnaire (SWAL-QOL). Health Qual Life Outcomes.

[16] Sasun A, Qureshi MI, Raghuveer R, Harjpal P (2023). Effectiveness of pelvis and trunk stabilization exercises over conventional physiotherapy to improve dynamic trunk balance in cerebellar ataxia: a randomized controlled trial. F1000Res.

[17] Jung YJ, Kim HJ, Choi JB, Park JS, Hwang NK (2020). Effect of dysphagia rehabilitation using kinesiology taping on oropharyngeal muscle hypertrophy in post-stroke patients: a double blind randomized placebo-controlled trial. Healthcare (Basel).

[18] Ayvat E, Onursal Kılınç Ö, Ayvat F, Savcun Demirci C, Aksu Yıldırım S, Kurşun O, Kılınç M (2022). The effects of exergame on postural control in individuals with ataxia: a rater-blinded, randomized controlled, cross-over study. Cerebellum.

[19] Keage M, Delatycki M, Corben L, Vogel A (2015). A systematic review of self-reported swallowing assessments in progressive neurological disorders. Dysphagia.

